# Prioritising candidate genes causing QTL using hierarchical orthologous groups

**DOI:** 10.1093/bioinformatics/bty615

**Published:** 2018-09-08

**Authors:** Alex Warwick Vesztrocy, Christophe Dessimoz, Henning Redestig

**Affiliations:** 1 Department of Genetics, Evolution and Environment, University College London, London, UK; 2 SIB Swiss Institute of Bioinformatics, Lausanne, Switzerland; 3 Department of Computational Biology, University of Lausanne, Lausanne, Switzerland; 4 Department of Computer Science, University College London, London, UK; 5 Centre for Integrative Genomics, University of Lausanne, Lausanne, Switzerland; 6 Bayer CropScience NV, Ghent, Belgium

## Abstract

**Motivation:**

A key goal in plant biotechnology applications is the identification of genes associated to particular phenotypic traits (for example: yield, fruit size, root length). Quantitative Trait Loci (QTL) studies identify genomic regions associated with a trait of interest. However, to infer potential causal genes in these regions, each of which can contain hundreds of genes, these data are usually intersected with prior functional knowledge of the genes. This process is however laborious, particularly if the experiment is performed in a non-model species, and the statistical significance of the inferred candidates is typically unknown.

**Results:**

This paper introduces QTLSearch, a method and software tool to search for candidate causal genes in QTL studies by combining Gene Ontology annotations across many species, leveraging hierarchical orthologous groups. The usefulness of this approach is demonstrated by re-analysing two metabolic QTL studies: one in *Arabidopsis thaliana*, the other in *Oryza sativa* subsp. *indica*. Even after controlling for statistical significance, QTLSearch inferred potential causal genes for more QTL than BLAST-based functional propagation against UniProtKB/Swiss-Prot, and for more QTL than in the original studies.

**Availability and implementation:**

QTLSearch is distributed under the LGPLv3 license. It is available to install from the Python Package Index (as qtlsearch), with the source available from https://bitbucket.org/alex-warwickvesztrocy/qtlsearch.

**Supplementary information:**

Supplementary data are available at *Bioinformatics* online.

## 1 Introduction

Identification of variants of genes that are linked to differences in phenotypic traits is a first step in many plant biotechnology applications. By creating mapping populations, characterizing and genotyping the individuals of these, it is often possible to find trait-associated regions of chromosomes—so-called Quantitative Trait Loci (QTL). However, a single QTL can typically contain hundreds, if not thousands, of genes. Thus, from a single study, it is rarely straight-forward to pinpoint the causal gene (if there is one at all) and multiple evidence is typically required.

Wide QTL can be broken down by performing additional experiments using higher-resolution genetic maps. A faster complementary approach is to annotate the genes in the target species with known associations to the trait of interest (for example, involvement in relevant pathways or biological processes), and searching for overlap with the genes inside a given QTL (Bargsten *et al.*, 2014; Chen *et al.*, 2012; Gong *et al.*, 2013; Lisec *et al.*, 2009). This approach has aided the identification of several verified causal genes—for example, the AT5G50950 fumarase (Brotman *et al.*, 2011; Lisec *et al.*, 2008)— demonstrating its merit.

Propagating gene-function annotations across and within species whilst taking evolutionary distance into account, alongside ensuring to control for chance co-occurrence, is difficult. This is particularly the case for non-model species that may have little or no curated annotations available. Currently, there are no dedicated tools to facilitate this analysis, potentially leading important insight to be missed.

**Fig. 1.**
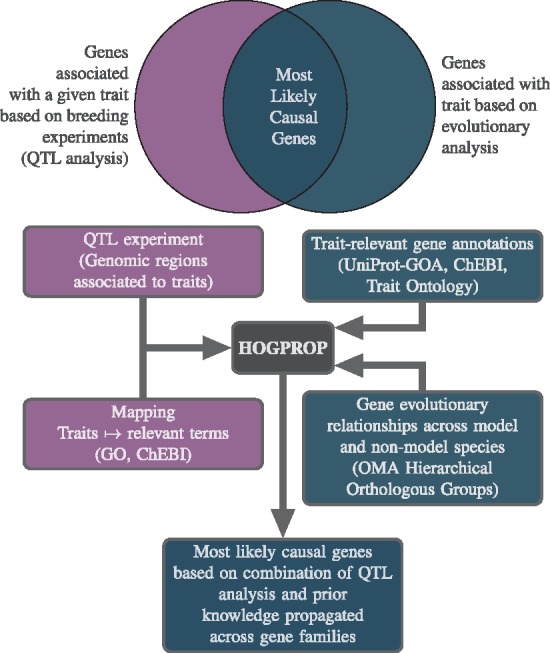
Conceptual overview of QTLSearch—to identify the most likely causal genes, by identifying the intersection of genes associated with a given trait based on an evolutionary analysis and QTL analyses

This paper presents QTLSearch—a method and tool which aims to recommend genes that are plausible candidates for causing an observed QTL, by identifying the intersection of those associated with a given trait based on an evolutionary analysis and one or more QTL analyses (Fig. 1) . That is, QTLSearch is a method for integrating data from public resources (for example, as Gene Ontology [GO] annotations) with the genomic regions identified during a QTL experiment. Gene families, in the form of hierarchical orthologous groups from the Orthologous MAtrix project (OMA) (Altenhoff *et al.*, 2018), enable reasoning over complex nested homologies in a consistent framework. By integrating functional inference with homology mapping, it is possible to differentiate the confidence in orthologous and paralogous relationships when propagating functional knowledge.

This method takes existing functional annotations (in an ontology-aware manner). As such, traits measured in QTL experiments need to be mapped to relevant terms. For instance, if the trait of interest was an abundance of the metabolite *Galactose*, this could be mapped to the GO term for ‘Galactose bio-synthetic process’ (GO:0046369), as well as to the ChEBI term for *Galactose* (CHEBI:28260). Existing gene annotations to this GO and ChEBI term would then be mapped to the trait and propagated through hierarchical orthologous groups, using the HOGPROP algorithm.

This propagated knowledge is then used to find genes, with an evidence trail, that are located in QTL for a given trait and homologous with another gene, possibly in a different species, that via functional annotations is known to be associated with that same trait.

While QTLSearch is applicable to any type of QTL studies, this paper shall demonstrate the usefulness of this method using two metabolic QTL studies in *Oryza sativa* subsp. *indica* from Gong *et al.* (2013) and *Arabidopsis thaliana* from Lisec *et al.* (2009), each reporting several QTL for a large number of metabolite abundances (phenotypic traits). This shows that QTLSearch can find similar results to those found in the more manual efforts, reported in the original studies. Furthermore, it also provides additional insight which was not reported in those studies.

## 2 Materials and methods

QTLSearch is underpinned by the HOGPROP algorithm, which uses the hierarchical orthologous groups from the OMA project in order to predict GO terms. The framework has been extended to permit propagation of general gene-labels (traits), resulting in a per-label score for each gene. This section starts with a high-level description of the HOGPROP algorithm, before describing the additions required to implement QTLSearch. The section then ends with a description of the datasets and method of comparison.

### 2.1 HOGPROP—gene annotation propagation and inference

**Fig. 2.**
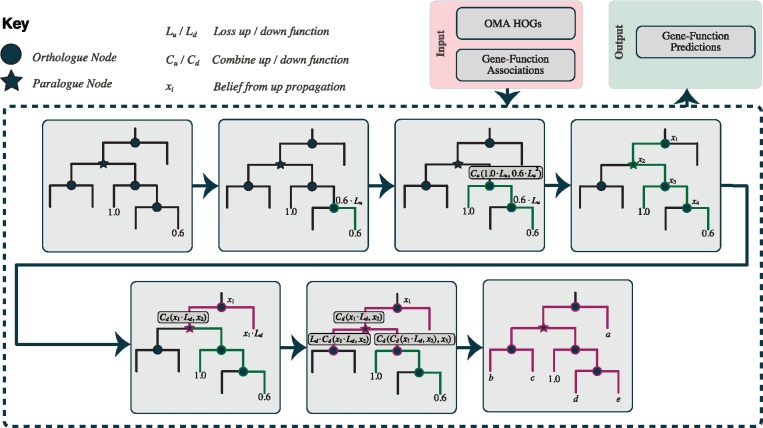
Overview of the HOGPROP algorithm, for propagating through hierarchical orthologous groups. This visualizes the propagation of a single gene-function association

Propagating functional annotations along gene phylogenies is a classical notion [for example, Eisen (1998)]. However, reconstructing large gene trees remains computationally demanding and error-prone. As a more scalable alternative, annotations can be propagated across hierarchical orthologous groups (HOGs) (Sonnhammer *et al.*, 2014). For instance, in the case of GO annotations, a subset {experimental and some electronic annotations [based on Škunca *et al.* (2012)]} are given a score dependent on their evidence code. These terms, with scores, are then associated with the leaves of the hierarchical structure (genes), before being pushed up and pulled down the hierarchy as can be seen in Figure 2. The score decays across each edge, currently set at a fixed rate of 20%, with a penalty when propagating over paralogous relationships of a double decay. Scores are combined at each node (using summation) during the up-propagation, whilst the maximum score is taken in the down-propagation. This is performed in an ontology-aware manner. That is, when dealing with ontology-based knowledge, the score associated to a particular term is also relevant to all terms less specific (parent terms) in the ontology.

After propagation, a score is available for every input annotation on all genes that are members of a group. This algorithm, termed ‘HOGPROP’, has previously been submitted to the second CAFA experiment (team name ‘CBRG’), where it performed well under several criteria (Jiang *et al.*, 2016). The algorithm shall be described in further detail, alongside in-depth benchmarking in a forthcoming publication.

### 2.2 Required adaptations to the original HOGPROP algorithm

This section will look at each of the adaptations required, in turn, to re-purpose the HOGPROP algorithm to search for trait-associated genes.

#### 2.2.1 Scoring

Let a single QTL be defined simply by its coordinates. That is, the triple (*C*, *s*, *e*), where *s* and *e* denote the start and end positions on the chromosome (*C*) of the QTL, respectively. If a chromosome is of *n* genes in length, it shall be denoted as a set of *n* genes. That is,

$$C:=\{g_{i}:1\leq i\leq n\}.$$

The genes that lie completely, or partially, within a QTL are then defined as

$$Q_{\left(s,e\right)}^{C}:=\{g_{i}:g_{i}\in C,s<g_{i}^{\text{end}},e>g_{i}^{\text{start}}\},$$

where $g_{i}^{\text{start}}$ and $g_{i}^{\text{end}}$ are the start and end positions of gene *g_i_*.

Then, let the score associated to a particular gene at time *t* be denoted as $S_{g_{i}}^{t}$. Initially (i.e. at *t *=* *0), each gene within a QTL is associated with the trait of interest with a uniform scoring, of

$$S_{g_{i}}^{0}:=\frac{1}{\left|Q\right|}.$$

Functional annotations can be given as input to the HOGPROP algorithm with varying initial scores. For example, in the case of the UniProt-GOA, experimentally derived annotations are currently set at an initial score of 1, whilst ‘trusted’ electronic annotations [based on Škunca *et al.* (2012), see Supplementary Table S1] are given a score of 0.95.

For each QTL, individually, these scores are associated with the genes, at the leaves of the HOGs. The scores are then propagated up and down the hierarchy, after-which (i.e. at *t *=* *1) the observed score increase for each gene in the QTL,

$$\Delta S_{g_{i}}=S_{g_{i}}^{1}-S_{g_{i}}^{0}=S_{g_{i}}^{1}-\frac{1}{\left|Q\right|},$$

is stored. This reflects the uniform probability of causal trait-association under the assumption that variation in a single gene is resulting in the observed QTL. This then gives an ordering to the genes in a particular QTL, to which extent they are associated with the trait of interest.

#### 2.2.2 Controlling for significance

A large QTL has a much greater chance to randomly overlap with genes with direct annotations, or have a close homologue with a relevant labelling. The narrower a QTL is, the smaller the chance of a spurious coincidence between a QTL and genes annotated as relevant for a given trait.

**Fig. 3.**
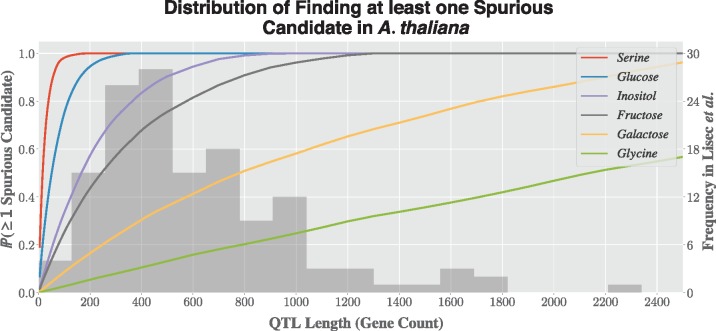
Probability of finding at least one spurious candidate in *A. thaliana* for six metabolites, as a function of QTL length (left *y*-axis). In the background, histogram of the distribution of QTL lengths reported by Lisec *et al.* (2009) (right *y*-axis)

In order to illustrate this issue, genes in *A. thaliana* (Ensembl Plants 20/TAIR10) were annotated with association of the abundance of six metabolites (the traits) using annotations to the GO and cross references between UniProtKB and ChEBI terms, listed in Table 1. Looking at every possible sliding window, for window sizes varying from just five genes up to 2500 genes, the number of times at least one gene is associated with the trait was computed. It shows that for typical QTL lengths, the probability of finding at least one spurious candidate can be substantial (Fig. 3).

**Table 1.** The six metabolites and their mapped GO and ChEBI terms used to find the distribution of finding at least one spurious candidate in *A. thaliana*

Metabolite	GO term	ChEBI term
*Serine*	GO:0006564	CHEBI:17822
*Glucose*	GO:0006094	CHEBI:17234
*Inositol*	GO:0006021	CHEBI:24848
*Fructose*	GO:0046370	CHEBI:24848
*Galactose*	GO:0046369	CHEBI:28260
*Glycine*	GO:0006545	CHEBI:15428

To account for this, QTLSearch can compute an empirical distribution of score increases per QTL-trait pairing, through the randomization of the coordinates of the QTL. The sampling of the coordinates is based on gene-count—both the chromosome and location on the locus are sampled. This feature gives the ability to report empirical *P*-values, which enable the control of significance. If the *P*-value estimation is enabled, by default the number of resamples is set to 1000.

When the aim of the QTL study is to search for candidate genes for a given trait among several QTL, it additionally becomes important to correct for the increase of false positive gene-trait associations. While the distribution of score-increases under the null-hypothesis depends strongly on the distribution and number of trait-associated genes, both of which are fixed, the tests become dependent meaning correction for multiple testing is not straight-forward. Leaving the investigation of a more suitable approach for a future study, tests reported here are corrected for falsely reporting at least one significant gene-trait association, i.e. the smallest *P*-value from each QTL, using Benjamini-Hochberg false discovery rate adjusted *P*-values (Benjamini and Hochberg, 1995). The unadjusted shall be denoted as *P*, with those adjusted as $P_{\text{BH}}$. The adjusted *P*-values were computed using the p. adjust function in R.

#### 2.2.3 Software package

QTLSearch is implemented as a Python package and is freely distributed under the LGPLv3 license, requiring Python 3.6 or later. It has been published on the Python Package Index (PyPI). Thus, it is installable using pip by issuing the command

pip install qtlsearch

The source code is available from https://bitbucket.org/alex-warwickvesztrocy/qtlsearch. As the software has been published under an open-source license, it is possible to add extra parsers for alternative data-sources with relative ease.

### 2.3 Datasets

To demonstrate the usefulness of QTLSearch, two datasets from metabolic QTL studies (Gong *et al.*, 2013; Lisec *et al.*, 2009) have been used. The dataset from Lisec *et al.* contains 141 QTL (with full coordinates) linked to 50 different metabolites in *A. thaliana*, whilst the Gong *et al.* dataset consists of 1260 QTL linked to the abundance of 302 metabolites in *O. sativa* subsp. *indica*. However, co-ordinates (as well as the authors’ predictions) were based on *O. sativa* subsp. *japonica*.

Hierarchical orthologous groups were taken from the September 2014 release of OMA, so that the MSU version 6 of *O. sativa* subsp. *japonica* was included. The UniProt-GOA (Barrell *et al.*, 2009) release from February 2018 was used, alongside the GO definition from 25th March 2018 (Ashburner *et al.*, 2000; Gene Ontology Consortium, 2017). External references from the ChEBI to UniProt entries were taken from ChEBI release 161 (Hastings *et al.*, 2016).

QTLSearch requires a mapping of the GO and ChEBI terms to map to the trait of interest, in this case the relevant metabolites. For initial scores originating from functional annotations in the UniProt-GOA database, initial scores are set at 1.0 for experimentally derived annotations and 0.95 for certain electronic annotations. [Electronic annotations (IEA evidence code) are filtered based on Škunca et al. (2012). See Supplementary Table S1 for filtering used.] Those arising from a cross-reference to the ChEBI are included with an initial score of 1. Genes with multiple sources are given the maximum of the initial scores.

Many of the metabolites measured in the studies could not straight-forwardly be mapped to a GO term, so some were mapped to more general (however, still relevant) terms. ChEBI associations were only included when an exact match to the compound was possible. For the mapping between metabolic traits and GO and/or ChEBI terms used, see Supplementary Tables S2 and S3. Table 2 shows the proportion of metabolites and QTL that have been mapped from each of the studies.

**Table 2.** Statistics of the number of QTL that could be mapped to GO and/or ChEBI terms from the two datasets in *A. thaliana* (Lisec *et al.*, 2009) and *O. sativa* subsp. *indica* (Gong *et al.*, 2013)

	*Author reported*		*Mapped to GO / ChEBI*	
Dataset	Metabolites	QTL	Metabolites	QTL
Lisec *et al.* (2009)	50	141	35	107
Gong *et al.* (2013)	302	1, 260	121	638

### 2.4 Comparison method—naïve BLAST

As well as comparing QTLSearch to the candidates that the respective authors reported, a comparison in performance was made to a naïve BLAST method. This takes the protein sequence for every gene inside the QTL and performs a BLAST against the entire UniProtKB/Swiss-Prot database [The UniProt Consortium (2017a); February 2018 release], using the NCBI BLAST+ tool (Camacho *et al.*, 2009) and the GNU Parallel tool in order to exploit parallelism in the search (Tange, 2011).

Candidate genes are predicted, as potentially causal to the abundance of a metabolite, if any of the top 10 hits, with an E-value of below $10^{-6}$ has a GO annotation (in the UniProt-GOA database [Barrell *et al.*, 2009; February 2018 release]) or cross-reference to a relevant ChEBI term, which is included in the mapping of metabolite to GO/ChEBI terms. Other E-value cut-offs ($10^{-3},\;10^{-9},\;10^{-12}$) gave similar results in this study. Further, the GO annotations are filtered to the same level as for QTLSearch.

## 3 Results

To illustrate the usefulness of QTLSearch, data from two previous metabolic QTL studies was re-analysed—one in *A. thaliana* (Lisec *et al.*, 2009), the other in *O. sativa* subsp. *indica* (Gong *et al.*, 2013)—in which candidate causal genes were identified for a subset of the QTL using ad hoc methods. First, aggregate results are presented, before looking at an example from each of these datasets.

### 3.1 Number of predictions

**Fig. 4.**
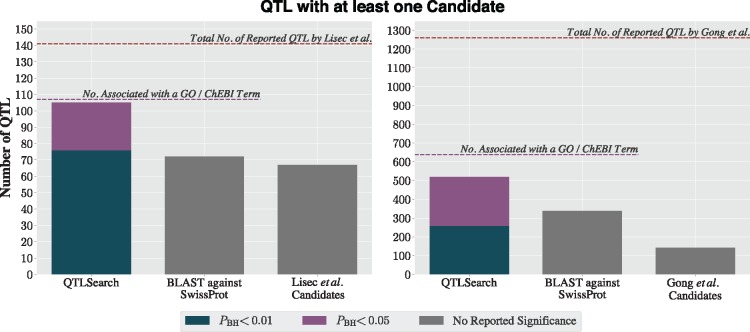
Proportion of QTL with at least one candidate from Lisec *et al.* (left) and Gong *et al.* (right) for each method

Lisec *et al.* identified 141 QTL. For 67 of these, they inferred at least one candidate gene. In comparison, QTLSearch was able to identify at least one candidate gene for 76 QTL with $P_{\text{BH}}<0.01$ (85 for *P *<* *0.01), and a further 29 QTL when relaxing the significance to $P_{\text{BH}}<0.05$ (20 for *P *<* *0.05)—see Figure 4. However, the BLAST against UniProtKB/Swiss-Prot identified a candidate gene for 72 QTL. The limiting factor for QTLSearch was the number of metabolites which could be associated to GO or ChEBI terms (available for 107 of the 141 QTL).

In the study by Gong *et al.*, 1260 QTL were identified with the authors inferring at least one candidate gene for 142 QTL. This lower proportion was likely due to the practical difficulties of analysing a much larger set of QTL using a labour-intensive ad hoc approach. By contrast, on this dataset, QTLSearch identified candidate genes for substantially more QTL than the original study (259 with $P_{\text{BH}}<0.01$ [360 for *P *<* *0.01] and 518 with $P_{\text{BH}}<0.05$ [same for *P *<* *0.01]; Fig. 4). The naïve BLAST search also performed much better for this dataset (338 QTL), finding candidate genes for a comparable number of QTL as QTLSearch, albeit without control for significance. Again, the limiting factor lies in the number of metabolites that could be associated with GO terms, which capped the number of QTL possible to predict using these methods to 638 out of 1260.

### 3.2 Overlap in predictions with original studies

**Fig. 5.**
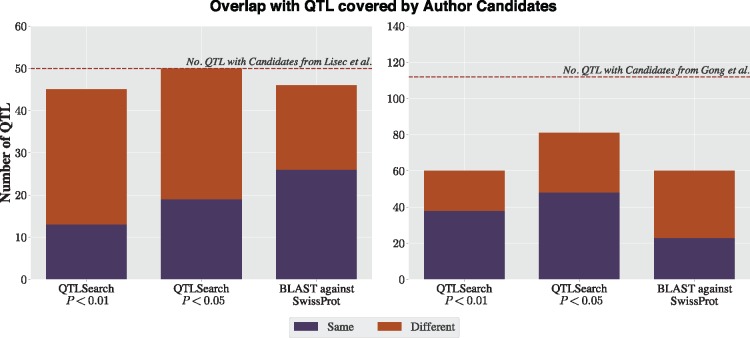
Overlap with the candidate genes reported by Lisec *et al.* (left) and Gong *et al.* (right), for QTLSearch (at 1% and 5% significance levels) and the naïve BLAST method

An assessment of the overlap between predictions from the original studies and the two automated approaches was also performed (Fig. 5). Authors of both studies gave multiple candidates for a subset of the QTL they reported. Here, the overlap is determined based on if a method predicted at least one of these. However, both QTLSearch and the BLAST method may have predicted more candidate genes than this.

When looking at the Lisec *et al.* dataset, both QTLSearch and BLAST find a candidate for the majority of the QTL, with QTLSearch finding a candidate for all when relaxing to the 5% level. BLAST agrees with the authors for half of the QTL. However, there is substantial disagreement in the predicted candidate genes for both methods.

As for the Gong *et al.* dataset, the authors reported either one or two candidates per QTL, with many having two candidates. QTLSearch only finds a candidate for just over half of the QTL which Gong *et al.* gave a prediction, at the 1% level (Fig. 5). The proportion increases to roughly two thirds at the 5% level. There is also substantial disagreement in the predicted candidate genes. A similar picture emerges when comparing the BLAST results to the original authors’ predictions.

### 3.3 Examples

In the dataset from Lisec *et al.*, there is a QTL associated with the abundance of *Galactose* which is approximately 2.3 Mbp in length, containing 309 genes. This particular metabolite was associated with both the ‘Galactose bio-synthetic process’ (GO:0046369) GO term, as well as to the ChEBI term for *Galactose* (CHEBI:28260).

**Fig. 6.**
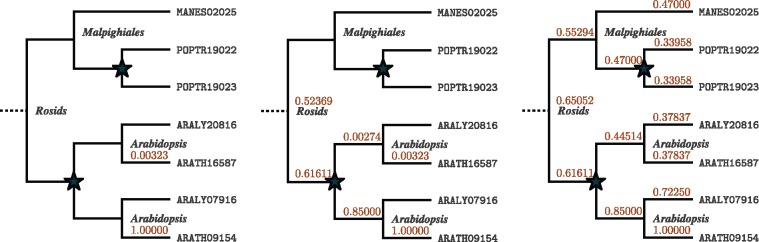
Visualization of the propagation of the annotation of ARATH09154 to CHEBI :28260 (*Galactose*), which leads to an increase in the score for ARATH16587. (Left) before propagation; (Middle) after up-propagation; (Right) After both up-propagation and down-propagation. *Note*: this hierarchical orthologous group extends above the level of the *Rosids*

There were no predictions for this particular QTL from the authors, however QTLSearch finds two results with *P *<* *0.01—see Table 3. The first of these (ARATH16826) has a direct annotation in the ChEBI and is also found by the naïve BLAST method described in Section 2.4. The second, ARATH16587, is not. This OMA identifier maps to the UniProtKB entry Q9SBA7, which has a recommended protein name of ‘Sugar transport protein 8’ (The UniProt Consortium, 2017b). Figure 6 shows the propagation from ARATH09154, which leads to the increase in score for ARATH16587.

**Table 3.** Table of significantly associated genes for a QTL in the Lisec *et al.* dataset, associated with *Galactose*

	QTLSearch				
OMA ID	Increase	*P*-value	Direct Annotation	Found by BLAST	Author Candidate
ARATH16826	0.996764	0.003126	ChEBI	✓	✗
ARATH16587	0.375134	0.003916	✗	✗	✗

Gong *et al.* associated a region approximately 1.03 Mbp in length, containing 146 genes with the abundance of *Chrysoeriol c-hexoside* (a flavanoid). As the GO is not particularly detailed in this area, this was associated with the generic ‘Flavonoid biosynthetic process’ (GO:0009813) GO term.

All candidate causal genes, reported by QTLSearch (with *P *<* *0.01) are located in the same hierarchical orthologous group (HOG:0164195) —see Table 4. These are all listed as ‘Chalcone and stilbene synthases’ in their relevant UniProtKB entries (The UniProt Consortium, 2017a), which catalyse the first committed step in the flavonoid synthesis pathway (Tohge *et al.*, 2007).

**Table 4.** Significantly associated genes for a QTL in the Gong *et al.* dataset, associated with *Chrysoeriol c-hexoside*

	QTLSearch				
OMA ID	Increase	*P*-value	Direct Annotation	Found by BLAST	Author Candidate
ORYSJ56351	1.980263	0.000021	✗	✗	✗
ORYSJ56362	1.494041	0.000048	UniProt-GOA	✗	✓
ORYSJ56358	0.638598	0.000260	✗	✓	✗
ORYSJ56359	0.638598	0.000260	✗	✓	✓
ORYSJ56355	0.541781	0.000418	✗	✓	✗

Only three of these five were found by the naïve BLAST method, with only one having a direct annotation in UniProt-GOA.

## 4 Discussion

QTLSearch provides a method for identifying the intersection of genes associated with a given trait based on an evolutionary analysis and QTL analyses. The hierarchical orthologous groups from OMA are at the centre of this, providing a consistent framework to reason over complex nested homologies. Instead of the potentially painstaking manual efforts usually required, QTLSearch provides a prioritized list of candidate genes causing the QTL by integrating annotation data, potentially from many sources.

It is clear that QTLSearch has the ability to predict potentially causal genes for many of the QTL reported in the studies used, especially when accepting at the nominal 5% significance level. Despite this, the naïve BLAST method (described in Section 2.4) appears to overlap further with the candidates predicted by Lisec *et al.* However, BLAST is simply a search to the most similar gene, whereas QTLSearch is able to take into account the fine-grained evolutionary history encoded inside the hierarchical orthologous groups. When more than one gene is predicted by QTLSearch, this enables the ordering of these based on the evidence trail. Further, the BLAST method does not take into account the probability of homology with genes with a direct annotation, shown in Section 2.2.2 to be more of an issue than may be expected.

For both of the datasets, QTLSearch predicts at least one candidate gene for more QTL than the naïve BLAST method. Experimental validation of these would be costly. However, the examples shown in Section 3.3 give plausibility to the results.

QTLSearch heavily relies on the existence of functional annotations and a map between these and the metabolites in question. Functional annotations can either be direct annotations to the species in the QTL analysis, or to closely related species. However, if there are no high-quality experimental annotations it is unlikely that either method will give useful results.

When considering the Lisec *et al.* dataset, it rapidly became clear that there were too few GO annotations at an acceptable level of evidence. This motivated the inclusion of the ChEBI as an additional source of information. The mapping performed between ChEBI and the metabolites that was adopted is however keyword-based and thus quite coarse. For instance, many of the cross-references from ChEBI for *Serine* are likely to be to *serine protein kinase*, which would be irrelevant to the question at hand. Refining the mapping should improve further the performance of QTLSearch for the metabolite QTL use-case. Similarly, it would be possible to extend the framework to include biological pathway information from databases such as Reactome (Fabregat *et al.*, 2018) or KEGG (Kanehisa *et al.*, 2017), possibly in an automated manner. However, this inclusion of knowledge from the ChEBI has meant that rather than simply loading GO annotations, the parser has been designed to be modular. Due to the open-source license, this enables easier inclusion of annotations from further sources.

Looking beyond metabolite QTL studies, agronomical or physiological traits for plants or animals alike, could also be analysed using QTLSearch by generating databases of genes that are associated with traits using, for example, the Trait Ontology (TO) (Shrestha *et al.*, 2012), before searching for co-incidence between QTL and genes homologous to genes in those lists.

Here, just as in the metabolite QTL setting, the use of ontologies is attractive. Instead of manually having to keep track of the relationship between terms, for example, ‘kernel size’ and ‘fruit size’ or ‘branched chain amino acid biosynthesis’ and ‘valine biosynthesis’, the ontology provides the necessary ‘is a’ relationships in order to directly use both annotations in an appropriate manner.

Likewise, this framework could also accommodate additional types of data, such as gene expression data. In the context of human genetics, several tools have been recently introduced to integrate expression alongside annotations (Arnold *et al.*, 2015; Stacey *et al.*, 2017; Watanabe *et al.*, 2017). These frameworks, however, do not naturally extend to other species. For plants, the possibility to include gene expression data is particularly interesting as it provides a straight-forward means to add prior knowledge to the nature of the causal gene(s). For example, via a grafting experiment it may be known that the sought gene is expressed in a given tissue, and are therefore searching for genes in a QTL for given trait *and* annotated to certain biological processes *and* expressed in that tissue.

One limitation of QTLSearch that hampers the use of continuous data such as gene expression is that the current scoring mechanism in the propagation algorithm is not probabilistic, and as such the confidence values propagated along the hierarchical orthologous groups are not directly interpretable. Adoption of a probabilistic method similar to Engelhardt *et al.* (2011) is planned. Meanwhile, results from the second CAFA (Jiang *et al.*, 2016), as well as preliminary results from the third CAFA, have shown that the current scoring method is competitive in the field of GO prediction.

Another limitation lies in the relatively high computational cost of estimating *P*-values, which is currently implemented as a permutation test. The runtime scales approximately linearly with the number of resamples required (default of 1000). This means that most of the time is spent on computing the empirical distribution. It may be possible to parameterize this, which would greatly decrease runtime. Meanwhile, it is possible to skip computation of the empirical distribution, which will still result in an ordered list of candidates.

Nevertheless, already in its current form, QTLSearch is a compelling alternative to the ad hoc approaches of typical QTL studies in plants. A fully automated framework also has clear advantages in terms of reproducibility.

## Supplementary Material

Supplementary Data

## References

[bty615-B1] Altenhoff A.M. et al (2018) The OMA orthology database in 2018: retrieving evolutionary relationships among all domains of life through richer web and programmatic interfaces. Nucleic Acids Res., 46, D477–D485. 29106550 10.1093/nar/gkx1019 PMC5753216

[bty615-B2] Arnold M. et al (2015) SNiPA: an interactive, genetic variant-centered annotation browser. Bioinformatics, 31, 1334–1336. 25431330 10.1093/bioinformatics/btu779 PMC4393511

[bty615-B3] Ashburner M. et al (2000) Gene ontology: tool for the unification of biology. Nat. Genet., 25, 25. 10802651 10.1038/75556 PMC3037419

[bty615-B4] Bargsten J.W. et al (2014) Prioritization of candidate genes in QTL regions based on associations between traits and biological processes. BMC Plant Biol., 14, 330. 25492368 10.1186/s12870-014-0330-3 PMC4274756

[bty615-B5] Barrell D. et al (2009) The GOA database in 2009—an integrated Gene Ontology Annotation resource. Nucleic Acids Res., 37, D396–D403. 18957448 10.1093/nar/gkn803 PMC2686469

[bty615-B6] Benjamini Y., Hochberg Y. (1995) Controlling the false discovery rate: a practical and powerful approach to multiple testing. J. R. Stat. Soc. B, 57, 289–300.

[bty615-B7] Brotman Y. et al (2011) Identification of enzymatic and regulatory genes of plant metabolism through QTL analysis in Arabidopsis. J Plant Physiol, 168, 1387–1394. 21536339 10.1016/j.jplph.2011.03.008

[bty615-B8] Camacho C. et al (2009) BLAST+: architecture and applications. BMC Bioinformatics, 10, 421. 20003500 10.1186/1471-2105-10-421 PMC2803857

[bty615-B9] Chen C. et al (2012) PICARA, an analytical pipeline providing probabilistic inference about a priori candidates genes underlying genome-wide association QTL in plants. PLoS ONE, 7, e46596. 23144785 10.1371/journal.pone.0046596 PMC3492367

[bty615-B10] Eisen J.A. (1998) Phylogenomics: improving functional predictions for uncharacterized genes by evolutionary analysis. Genome Res., 8, 163–167. 9521918 10.1101/gr.8.3.163

[bty615-B11] Engelhardt B.E. et al (2011) Genome-scale phylogenetic function annotation of large and diverse protein families. Genome Res., 21, 1969–1980. 21784873 10.1101/gr.104687.109 PMC3205580

[bty615-B12] Fabregat A. et al (2018) The reactome pathway knowledgebase. Nucleic Acids Res., 46, D649–D655. 29145629 10.1093/nar/gkx1132 PMC5753187

[bty615-B13] Gene Ontology Consortium (2017) Expansion of the gene ontology knowledgebase and resources. Nucleic Acids Res., 45, D331–D338. 27899567 10.1093/nar/gkw1108 PMC5210579

[bty615-B14] Gong L. et al (2013) Genetic analysis of the metabolome exemplified using a rice population. Proc. Natl. Acad. Sci., 110, 20320–20325. 24259710 10.1073/pnas.1319681110 PMC3864304

[bty615-B15] Hastings J. et al (2016) ChEBI in 2016: improved services and an expanding collection of metabolites. Nucleic Acids Res., 44, D1214–D1219. 26467479 10.1093/nar/gkv1031 PMC4702775

[bty615-B16] Jiang Y. et al (2016) An expanded evaluation of protein function prediction methods shows an improvement in accuracy. Genome Biol., 17, 184. 27604469 10.1186/s13059-016-1037-6 PMC5015320

[bty615-B17] Kanehisa M. et al (2017) KEGG: new perspectives on genomes, pathways, diseases and drugs. Nucleic Acids Res., 45, D353–D361. 27899662 10.1093/nar/gkw1092 PMC5210567

[bty615-B18] Kreft Ł. et al (2017) PhyD3: a phylogenetic tree viewer with extended phyloXML support for functional genomics data visualization. Bioinformatics, 33, 2946–2947. 28525531 10.1093/bioinformatics/btx324

[bty615-B19] Lisec J. et al (2008) Identification of metabolic and biomass QTL in Arabidopsis thaliana in a parallel analysis of RIL and IL populations. Plant J., 53, 960–972. 18047556 10.1111/j.1365-313X.2007.03383.x PMC2268983

[bty615-B20] Lisec J. et al (2009) Identification of heterotic metabolite QTL in Arabidopsis thaliana RIL and IL populations. Plant J., 59, 777–788. 19453458 10.1111/j.1365-313X.2009.03910.x

[bty615-B21] Shrestha R. et al (2012) Bridging the phenotypic and genetic data useful for integrated breeding through a data annotation using the crop ontology developed by the crop communities of practice. Front. Physiol., 3, 326. 22934074 10.3389/fphys.2012.00326 PMC3429094

[bty615-B22] Škunca N. et al (2012) Quality of computationally inferred gene ontology annotations. PLoS Comput. Biol., 8, e1002533. 22693439 10.1371/journal.pcbi.1002533 PMC3364937

[bty615-B23] Sonnhammer E.L. et al The Quest for Orthologs Consortium. (2014) Big data and other challenges in the quest for orthologs. Bioinformatics, 30, 2993–2998. 25064571 10.1093/bioinformatics/btu492 PMC4201156

[bty615-B24] Stacey D. et al (2017). ProGeM: A framework for the prioritisation of candidate causal genes at molecular quantitative trait loci. *bioRxiv*, page 230094. 10.1093/nar/gky837 PMC6326795 30239796

[bty615-B25] Tange O. (2011) GNU parallel—the command-line power tool. Login USENIX Mag., 36, 42–47.

[bty615-B26] The UniProt Consortium (2017a) UniProt: the universal protein knowledgebase. Nucleic Acids Res., 45, D158–D169. 27899622 10.1093/nar/gkw1099 PMC5210571

[bty615-B27] The UniProt Consortium (2017b). UniProtKB—Q9SBA7 (STP8_ARATH). https://www.uniprot.org/uniprot/Q9SBA7. Accessed: 13-03-2018.

[bty615-B28] Tohge T. et al (2007) Phytochemical genomics in Arabidopsis thaliana: a case study for functional identification of flavonoid biosynthesis genes. Pure Appl. Chem., 79, 811–823.

[bty615-B29] Watanabe K. et al (2017) Functional mapping and annotation of genetic associations with FUMA. Nat. Commun., 8, 1826. 29184056 10.1038/s41467-017-01261-5 PMC5705698

