# Supplementary material for: Prioritising candidate genes causing QTL using hierarchical orthologous groups
Source: Bioinformatics. 2018 Sep 8;34(17):i612–9. doi: 10.1093/bioinformatics/bty615 (PMC6129274; doi:10.1093/bioinformatics/bty615)
Supplement: Supplementary Data [file bty615_supp.zip › bty615-suppl_data/supplementary.pdf]

Prioritising Candidate Genes Causing QTL using Hierarchical Orthologous Groups

Supplementary Data

Alex Warwick Vesztrocy, Christophe Dessimoz and Henning Redestig

S1 Gene Ontology Annotation Filtering

Table S1: Filtering of GO evidence codes, based on Škunca *et al.* (2012) with the addition of IBA / IBD evidence codes. Those listed are included as initial functional knowledge in HOGPROP / QTLSearch.

Evidence Code	Reference Code (if relevant)	Initial Score	Description
EXP		1.0	Inferred from Experimental
IDA		1.0	Inferred from Direct Assay
IPI		1.0	Inferred from Physical Interaction
IMP		1.0	Inferred from Mutant Phenotype
IGI		1.0	Inferred from Genetic Interaction
IEP		1.0	Inferred from Expression Pattern
IBA		0.95	Inferred from Biological aspect of Ancestor
IBD		0.95	Inferred from Biological aspect of Descendant
IEA	2	0.95	Gene Ontology annotation through association of InterPro records with GO terms.
IEA	3	0.95	Gene Ontology annotation based on Enzyme Commission mapping.
IEA	4	0.95	Gene Ontology annotation based on Swiss-Prot keyword mapping.
IEA	23	0.95	Gene Ontology annotation based on Swiss-Prot Subcellular Location vocabulary mapping.
IEA	37	0.95	Gene Ontology annotation based on manual assignment of UniProtKB keywords in UniProtKB/Swiss-Prot entries.

Continued on next page

Table S1 – *continued from previous page*

Evidence Code	Reference Code (if relevant)	Initial Score	Description
IEA	38	0.95	Gene Ontology annotation based on automatic assignment of UniProtKB keywords in UniProtKB/TrEMBL entries.
IEA	39	0.95	Gene Ontology annotation based on the manual assignment of UniProtKB Subcellular Location terms in UniProtKB/Swiss-Prot entries.
IEA	40	0.95	Gene Ontology annotation based on the automatic assignment of UniProtKB Subcellular Location terms in UniProtKB/TrEMBL entries.
IEA	42	0.95	Gene Ontology annotation through association of InterPro records with GO terms, accompanied by conservative changes to GO terms applied by UniProt.
IEA	45	0.95	Gene Ontology annotation based on UniProtKB/TrEMBL entries keyword mapping, accompanied by conservative changes to GO terms applied by UniProt.
IEA	46	0.95	Gene Ontology annotation based on UniProtKB/TrEMBL Subcellular Location vocabulary mapping, accompanied by conservative changes to GO terms applied by UniProt.

S2 Trait Mappings

Table S2: Mapping from metabolite to GO and / or ChEBI terms for the dataset from Lisec *et al.* (2009).

	GO		ChEBI	
Trait	*Term*	*Name*	*Term*	*Name*
4-aminobutyric acid	GO:0009449	gamma-aminobutyric acid biosynthetic process	CHEBI:16865	gamma-aminobutyric acid
alpha-tocopherol	GO:0010189	vitamin E biosynthetic process	CHEBI:22470	alpha-tocopherol
ascorbic acid	GO:0019853	L-ascorbic acid biosynthetic process	CHEBI:22652	ascorbic acid

Continued on next page

Table S2 – *continued from previous page*

Trait	GO *Term*	GO *Name*	ChEBI *Term*	ChEBI *Name*
aspartic acid	GO:0006532	aspartate biosynthetic process	CHEBI:22660	aspartic acid
beta-alanine	GO:0019483	beta-alanine biosynthetic process	CHEBI:16958	beta-alanine
cellobiose	GO:2000891	cellobiose metabolic process	CHEBI:17057	cellobiose
cholesterol	GO:0006695	cholesterol biosynthetic process	CHEBI:16113	cholesterol
citrulline	GO:0000052	citrulline metabolic process	CHEBI:18211	citrulline
fructose	GO:0046370	fructose biosynthetic process	CHEBI:28757	fructose
fructose 6-phosphate	GO:0046370	fructose biosynthetic process	CHEBI:88003	fructose 6-phosphate
fucosterol	GO:0016126	sterol biosynthetic process	CHEBI:27865	fucosterol
galactonic acid	GO:0034192	D-galactonate metabolic process	CHEBI:24149	galactonic acid
galactose	GO:0046369	galactose biosynthetic process	CHEBI:28260	galactose
glucose	GO:0006094	gluconeogenesis	CHEBI:17234	glucose
glucose 6-phosphate	GO:0006094	gluconeogenesis	CHEBI:14314	D-glucose 6-phosphate
glycerol	GO:0006114	glycerol biosynthetic process	CHEBI:17754	glycerol
glycerol 3-phosphate	GO:0006114	glycerol biosynthetic process		
glycine	GO:0006545	glycine biosynthetic process	CHEBI:15428	glycine
hydroxyproline	GO:0019472	4-hydroxyproline biosynthetic process	CHEBI:24741	hydroxyproline
inositol	GO:0006021	inositol biosynthetic process	CHEBI:24848	inositol
lysine	GO:0009085	lysine biosynthetic process	CHEBI:25094	lysine
methionine	GO:0071265	L-methionine biosynthetic process	CHEBI:16811	methionine
nicotinic acid	GO:1901849	nicotinate biosynthetic process	CHEBI:15940	nicotinic acid
phenylalanine	GO:0009094	L-phenylalanine biosynthetic process	CHEBI:28044	phenylalanine
proline	GO:0055129	L-proline biosynthetic process	CHEBI:26271	proline
raffinose	GO:0033529	raffinose biosynthetic process	CHEBI:16634	raffinose
salicylic acid	GO:0009697	salicylic acid biosynthetic process	CHEBI:16914	salicylic acid
serine	GO:0006564	L-serine biosynthetic process	CHEBI:17822	serine
sinapic acid (cis)	GO:0033497	sinapate biosynthetic process	CHEBI:76350	cis-sinapic acid
sinapic acid (trans)	GO:0033497	sinapate biosynthetic process	CHEBI:15714	trans-sinapic acid
sucrose	GO:0005986	sucrose biosynthetic process	CHEBI:17992	sucrose

Continued on next page

Table S2 – *continued from previous page*

Trait	GO<br>*Term*	 *Name*	ChEBI<br>*Term*	 *Name*
threonine	GO:0009088	threonine biosynthetic process	CHEBI:26986	threonine
trehalose	GO:0005992	trehalose biosynthetic process	CHEBI:27082	trehalose
tyrosine	GO:0006571	tyrosine biosynthetic process	CHEBI:18186	tyrosine
xylose	GO:0042842	D-xylose biosynthetic process	CHEBI:18222	xylose

Table S3: Mapping from metabolite to GO and / or ChEBI terms for the dataset from Gong *et al.* (2013).

Trait	GO<br>*Term*	 *Name*	ChEBI<br>*Term*	 *Name*
(+)-dehydrovomifoliol	GO:0016114	terpenoid biosynthetic process	CHEBI:4372	(6S)-dehydrovomifoliol
(+)-threo-9,10-dihydroxystearic acid	GO:0006633	fatty acid biosynthetic process	CHEBI:49254	(S,S)-9,10-dihydroxyoctadecanoic acid
12-hydroxyarachidonic acid	GO:0006633	fatty acid biosynthetic process	CHEBI:19138	12-HETE
16-hydroxy-hexadecanoic acid	GO:0006633	fatty acid biosynthetic process	CHEBI:55328	juniperic acid
2″, 6″-o-diacetyloninin	GO:0009813	flavonoid biosynthetic process		
24-hydroxytetracosanoic acid	GO:0006633	fatty acid biosynthetic process	CHEBI:76930	omega-hydroxytetracosanoic acid
3′, 4′, 5′-dihydrotricetin o-hexosyl-o-hexoside	GO:0009813	flavonoid biosynthetic process		
3, 5, 7-trihydroxy-6-methoxy-4′-prenyloxyflavone	GO:0009813	flavonoid biosynthetic process		
3-ketosphinganine	GO:0006633	fatty acid biosynthetic process		
4′-o-methylpuerarin	GO:0009813	flavonoid biosynthetic process		
4-geranyloxy-5-methyl coumarin	GO:0009805	coumarin biosynthetic process		
5-caffeoylquinic acid methyl ester	GO:0009699	phenylpropanoid biosynthetic process		
5-hydroxy-l-tryptophan	GO:0000162	tryptophan biosynthetic process	CHEBI:17780	5-hydroxy-L-tryptophan
6,8-dihydroxy-5,7-dimethoxycoumarin	GO:0009805	coumarin biosynthetic process		
6-prenylnaringenin	GO:0009813	flavonoid biosynthetic process	CHEBI:27566	6-prenylnaringenin

Continued on next page

Table S3 – *continued from previous page*

Trait	GO Term	GO Name	ChEBI Term	ChEBI Name
9,10-epoxy-18-hydroxy-octadecanoic acid	GO:0006633	fatty acid biosynthetic process		
akd 2b1	GO:0006633	fatty acid biosynthetic process		
acetosyringone	GO:0072391	phenol biosynthetic process	CHEBI:2404	acetosyringone
acteoside	GO:0072391	phenol biosynthetic process	CHEBI:132853	acteoside
aliarin	GO:0009813	flavonoid biosynthetic process		
apigenin c-pentoside	GO:0009813	flavonoid biosynthetic process	CHEBI:131755	apigenin C-pentoside
axillarin	GO:0009813	flavonoid biosynthetic process	CHEBI:2941	axillarin
ayanin	GO:0009813	flavonoid biosynthetic process		
c-hexosyl-c-pentosyl-apigenin	GO:0009813	flavonoid biosynthetic process		
c-hexosyl-apigenin o-caffeoylhexoside	GO:0009813	flavonoid biosynthetic process		
c-hexosyl-apigenin o-hexosyl-o-hexoside	GO:0009813	flavonoid biosynthetic process		
c-hexosyl-apigenin o-hexosyl-o-hexosyl-o-hexoside	GO:0009813	flavonoid biosynthetic process		
c-hexosyl-apigenin o-p-coumaroylhexoside	GO:0009813	flavonoid biosynthetic process		
c-hexosyl-chrysin o-feruloylhexoside	GO:0009813	flavonoid biosynthetic process		
c-hexosyl-chrysoeriol o-p-coumaroylhexoside	GO:0009813	flavonoid biosynthetic process		
c-hexosyl-chrysoeriol o-feruloylhexoside	GO:0009813	flavonoid biosynthetic process		
c-hexosyl-chrysoeriol o-hexoside	GO:0009813	flavonoid biosynthetic process		
c-hexosyl-luteolin o-hexoside	GO:0009813	flavonoid biosynthetic process		
c-hexosyl-luteolin o-p-coumaroylhexoside	GO:0009813	flavonoid biosynthetic process		
c-hexosyl-luteolin o-pentoside	GO:0009813	flavonoid biosynthetic process		
c-hexosyl-methylchrysoeriol	GO:0009813	flavonoid biosynthetic process		
c-hexosyl-naringenin o-hexosyl-o-hexoside	GO:0009813	flavonoid biosynthetic process		
c-hexosyl-naringenin o-p-coumaroylhexoside	GO:0009813	flavonoid biosynthetic process		
c-pentosyl-apeignin o-feruloylhexoside	GO:0009813	flavonoid biosynthetic process		

Continued on next page

Table S3 – *continued from previous page*

Trait	GO		ChEBI	
	Term	*Name*	*Term*	*Name*
c-pentosyl-apigenin o-rutinoside	GO:0009813	flavonoid biosynthetic process		
c-pentosyl-apigenin o-caffeoylhexoside	GO:0009813	flavonoid biosynthetic process		
c-pentosyl-apigenin o-hexoside	GO:0009813	flavonoid biosynthetic process		
c-pentosyl-apigenin o-p-coumaroylhexoside	GO:0009813	flavonoid biosynthetic process		
c-pentosyl-chrysoeriol o-feruloylhexoside	GO:0009813	flavonoid biosynthetic process		
c-pentosyl-chrysoeriol o-hexoside	GO:0009813	flavonoid biosynthetic process		
c-pentosyl-luteolin o-hexoside	GO:0009813	flavonoid biosynthetic process		
c-rhamnosyl-apigenin o-hexoside	GO:0009813	flavonoid biosynthetic process		
cafestol	GO:0009813	flavonoid biosynthetic process	CHEBI:3291	cafestol
caohuoside d	GO:0046246	terpene biosynthetic process		
chryso-obtusin-o-hexoside	GO:0009813	flavonoid biosynthetic process		
chrysoeriol 5-o-hexoside	GO:0009813	flavonoid biosynthetic process		
chrysoeriol 7-o-hexoside	GO:0009813	flavonoid biosynthetic process		
chrysoeriol c-hexoside	GO:0009813	flavonoid biosynthetic process		
chrysoeriol c-hexoside derivative	GO:0009813	flavonoid biosynthetic process		
chrysoeriol o-malonyhexoside	GO:0009813	flavonoid biosynthetic process		
chrysoeriol o-rutinoside	GO:0009813	flavonoid biosynthetic process		
crotonoside	GO:0042451	purine nucleoside biosynthetic process	CHEBI:3927	Crotonoside
cyanidin 3-o-pentoside	GO:0009718	anthocyanin-containing compound biosynthetic process		
cymarin	GO:0046246	terpene biosynthetic process	CHEBI:4037	Cymarin
daidzein o-hexoside	GO:0009813	flavonoid biosynthetic process		
delphinidin o-hexoside	GO:0009718	anthocyanin-containing compound biosynthetic process		
deoxyguanosine	GO:0009163	nucleoside biosynthetic process		
ephemeranthoside	GO:0046246	terpene biosynthetic process		

Continued on next page

Table S3 – *continued from previous page*

Trait	GO *Term*	*Name*	ChEBI *Term*	*Name*
epicatechin o-hexoside	GO:0009813	flavonoid biosynthetic process		
eriodictyol c-hexoside	GO:0009813	flavonoid biosynthetic process		
fructose 1,6-diphosphate	GO:0046370	fructose biosynthetic process		
gibberellin a12	GO:0009686	gibberellin biosynthetic process	CHEBI:30088	gibberellin A12
gibberellin a15	GO:0009686	gibberellin biosynthetic process	CHEBI:29590	gibberellin A15 (diacid form)
gibberellin a53	GO:0009686	gibberellin biosynthetic process	CHEBI:27433	gibberellin A53
kaempferol derivative	GO:0009813	flavonoid biosynthetic process	CHEBI:28499	kaempferol
kievitone	GO:0009813	flavonoid biosynthetic process	CHEBI:16832	kievitone
kolavic acid	GO:0046246	terpene biosynthetic process		
lpc(1-acyl 12:1)	GO:0045017	glycerolipid biosynthetic process		
lpc(1-acyl 14:1)	GO:0045017	glycerolipid biosynthetic process	CHEBI:67054	lysophosphatidylcholine 14:1
lpc(1-acyl 16:0)	GO:0045017	glycerolipid biosynthetic process	CHEBI:64563	lysophosphatidylcholine 16:0
lpc(1-acyl 16:1)	GO:0045017	glycerolipid biosynthetic process	CHEBI:64560	lysophosphatidylcholine 16:1
lpc(1-acyl 16:2)	GO:0045017	glycerolipid biosynthetic process	CHEBI:67055	lysophosphatidylcholine 16:2
lpc(1-acyl 18:0)	GO:0045017	glycerolipid biosynthetic process	CHEBI:64561	lysophosphatidylcholine 18:0
lpc(1-acyl 18:2)	GO:0045017	glycerolipid biosynthetic process	CHEBI:64549	lysophosphatidylcholine 18:2
lpc(1-acyl 20:4)	GO:0045017	glycerolipid biosynthetic process	CHEBI:64568	lysophosphatidylcholine 20:4
lpc(1-acyl 24:4)	GO:0045017	glycerolipid biosynthetic process		
leu-ala-gly-lys	GO:0006520	cellular amino acid metabolic process		
leu-pro	GO:0006520	cellular amino acid metabolic process	CHEBI:73580	Leu-Pro
n2, n2-dimethyguanosine	GO:0009163	nucleoside biosynthetic process		
nicotianamine	GO:0030418	nicotianamine biosynthetic process	CHEBI:25520	nicotianamine
o-acetyl-l-serine	GO:0006520	cellular amino acid metabolic process	CHEBI:17981	O-acetyl-L-serine
o-malonylhexoside derivative	GO:0009813	flavonoid biosynthetic process		
o-methylchrysoeriol o-hexoside	GO:0009813	flavonoid biosynthetic process		
o-methylquercetin o-hexoside	GO:0009813	flavonoid biosynthetic process		
o-methylapigenin c-hexoside	GO:0009813	flavonoid biosynthetic process		

Continued on next page

Table S3 – *continued from previous page*

Trait	GO Term	GO Name	ChEBI Term	ChEBI Name
o-methylapigenin c-pentoside	GO:0009813	flavonoid biosynthetic process		
o-methylnaringenin c-pentoside	GO:0009813	flavonoid biosynthetic process		
phytocassane a	GO:0046246	terpene biosynthetic process	CHEBI:72664	(+)-phytocassane A
phytocassane c	GO:0046246	terpene biosynthetic process	CHEBI:72668	(+)-phytocassane C
polygodial	GO:0006694	steroid biosynthetic process	CHEBI:8305	Polygodial
spinacetin	GO:0009813	flavonoid biosynthetic process		
succinyladenosine	GO:0046086	adenosine biosynthetic process	CHEBI:71169	succinyladenosine
sucrose	GO:0005986	sucrose biosynthetic process	CHEBI:17992	sucrose
tricin 4′-o-(syringyl alcohol)ether	GO:0009813	flavonoid biosynthetic process		
tricin 4′-o-(syringyl alcohol)ether o-hexoside	GO:0009813	flavonoid biosynthetic process		
tricin 4′-o-(syringyl alcohol)ether derivative	GO:0009813	flavonoid biosynthetic process		
tricin 5-o-hexoside	GO:0009813	flavonoid biosynthetic process		
tricin 7-o-hexoside	GO:0009813	flavonoid biosynthetic process		
tricin o-hexoside derivative	GO:0009813	flavonoid biosynthetic process		
tricin o-hexosyl-o-hexoside	GO:0009813	flavonoid biosynthetic process		
tricin o-malonylhexoside	GO:0009813	flavonoid biosynthetic process		
tricin o-malonylhexoside derivative	GO:0009813	flavonoid biosynthetic process		
tricin o-rhamnosyl-o-malonylhexoside	GO:0009813	flavonoid biosynthetic process		
tricin o-rutinoside	GO:0009813	flavonoid biosynthetic process		
tricin o-sinapoylpentoside	GO:0009813	flavonoid biosynthetic process		
tricin derivative	GO:0009813	flavonoid biosynthetic process		
tricin-o-glucoside derivative	GO:0009813	flavonoid biosynthetic process		
tricin-o-hexoside derivative	GO:0009813	flavonoid biosynthetic process		
vitamin a	GO:0035238	vitamin A biosynthetic process	CHEBI:12777	vitamin A
vitamin b2	GO:0009231	riboflavin biosynthetic process	CHEBI:17015	riboflavin
di-c,c-hexosyl-apigenin	GO:0009813	flavonoid biosynthetic process		

Continued on next page

Table S3 – *continued from previous page*

	GO		ChEBI	
Trait	*Term*	*Name*	*Term*	*Name*
di-c,c-hexosyl-apigenin derivative	`GO:0009813`	flavonoid biosynthetic process		
di-c,c-hexosyl-chrysoeriol	`GO:0009813`	flavonoid biosynthetic process		
di-c,c-hexosyl-luteolin	`GO:0009813`	flavonoid biosynthetic process		
di-c,c-pentosyl-apigenin	`GO:0009813`	flavonoid biosynthetic process		
di-c,c-pentosyl-luteolin	`GO:0009813`	flavonoid biosynthetic process		
